# Supplementary material for: Emotional Components of Pain Perception in Borderline Personality Disorder and Major Depression—A Repetitive Peripheral Magnetic Stimulation (rPMS) Study
Source: Brain Sci. 2020 Nov 24;10(12):905. doi: 10.3390/brainsci10120905 (PMC7761125; doi:10.3390/brainsci10120905)
Supplement: Supplementary file 1 [file brainsci-10-00905-s001.pdf]

Emotional Components of Pain Perception in Borderline Personality Disorder and Major Depression – a repetitive peripheral magnetic stimulation (rPMS) study

[1]Kathrin Malejko, [2]André Huss, [1]Carlos Schönfeldt-Lecuona, [1]Maren Braun, [1]Heiko Graf

[1]Department of Psychiatry and Psychotherapy III, University of Ulm, Germany

[2]Department of Neurology, University of Ulm, Germany

Table S1: Characteristics of patients with Borderline personality disorder (BPD) and comorbid major depression, patients with major depressive disorder (MD) without BPD and healthy controls (HC).

		**BPD (*n*=10)**	**MD (*n*=12)**	**HC (*n*=12)**
Age of onset NSSI:				
	< 20 years	7	0	0
	> 20 years	3	0	0
Years of NSSI:				
	< 5 years	3	0	0
	> 5 years	7	0	0
Frequency of NSSI:				
	Daily	2	0	0
	Weekly	8	0	0
Substance abuse history:				
	Alcohol	1	1	0
	Psychotropic drugs	1	0	0
Medication:				
	Antidepressants	8	12	0
	Mood stabilizers	5	0	0
	Antiepileptic drugs	5	0	0
	Antipsychotic drugs	5	4	0
	Antihypertensive drugs	3	0	0
	Benzodiazepines	0	4	0
Dissociation Tension scale (mean±SD)	Before rPMS	2.80±2.07	1.88±1.59	n.a.
	After rPMS	2.35±2.23	1.80±1.86	n.a.
BDI (mean±SD)		39.56±12.20	29.67±11.23	n.a.

NSSI=nonsuicidal self-injury; SD=standard deviation; n.a=not assessed
